# Decision making in healthy participants on the Iowa Gambling Task: new insights from an operant approach

**DOI:** 10.3389/fpsyg.2015.00391

**Published:** 2015-04-07

**Authors:** Peter N. Bull, Lynette J. Tippett, Donna Rose Addis

**Affiliations:** School of Psychology, The University of AucklandAuckland, New Zealand

**Keywords:** decision making, Iowa Gambling Task, operant psychology, sensitivity to reward and punishment, learning rate

## Abstract

The Iowa Gambling Task (IGT) has contributed greatly to the study of affective decision making. However, researchers have observed high inter-study and inter-individual variability in IGT performance in healthy participants, and many are classified as impaired using standard criteria. Additionally, while decision-making deficits are often attributed to atypical sensitivity to reward and/or punishment, the IGT lacks an integrated sensitivity measure. Adopting an operant perspective, two experiments were conducted to explore these issues. In Experiment 1, 50 healthy participants completed a 200-trial version of the IGT which otherwise closely emulated Bechara et al.'s (1999) original computer task. Group data for Trials 1–100 closely replicated Bechara et al.'s original findings of high net scores and preferences for advantageous decks, suggesting that implementations that depart significantly from Bechara's standard IGT contribute to inter-study variability. During Trials 101–200, mean net scores improved significantly and the percentage of participants meeting the “impaired” criterion was halved. An operant-style stability criterion applied to individual data revealed this was likely related to individual differences in learning rate. Experiment 2 used a novel operant card task—the Auckland Card Task (ACT)—to derive quantitative estimates of sensitivity using the generalized matching law. Relative to individuals who mastered the IGT, persistent poor performers on the IGT exhibited significantly lower sensitivity to magnitudes (but not frequencies) of rewards and punishers on the ACT. Overall, our findings demonstrate the utility of operant-style analysis of IGT data and the potential of applying operant concurrent-schedule procedures to the study of human decision making.

## Introduction

Life is like a game of cards. The hand that is dealt you represents determinism; the way you play it is free will.

(Jawaharlal Nehru).

Poor decision making, particularly in situations involving complexity (where choice alternatives have multiple reward and punishment dimensions which may conflict) or uncertainty (where rewards and punishers occur unpredictably), is associated with brain injury to ventromedial prefrontal cortex (VMPFC). The Iowa Gambling Task (IGT; Bechara et al., 1994) was designed to assess decision-making abilities in VMPFC patients under such conditions of complexity and uncertainty. Participants are instructed to maximize winnings while choosing repeatedly from four decks of playing cards that unpredictably yield wins and losses. Importantly, the contingencies of reward and punishment are counter-intuitively arranged so that the decks with higher wins ($100) result in a long-term net loss, while the decks with smaller wins ($50) yield a net gain. Participants who do not learn to prefer one or both of the $50 decks over the course of 100 trials are considered to exhibit a decision-making impairment. Over the last 20 years the IGT has become a de-facto standard for decision-making research (Dunn et al., 2006) and has been marketed as a tool for clinical assessment (Bechara, 2007). Indeed, the IGT has not only contributed to understanding decision-making deficits in patients with VMPFC damage, but has also been successfully applied to a variety of disorders arising from poor impulse control (e.g., pathological gambling; Brand et al., 2005).

**Figure 1:**
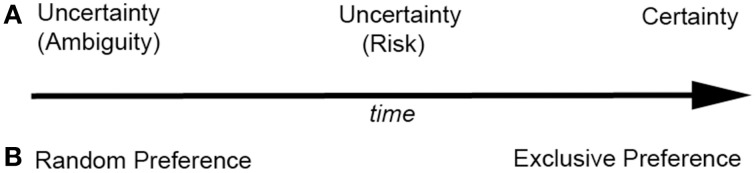
**Continuum representing the change in a participant's (A) level of certainty and (B) preferences in a decision-making task as a function of time**.

Theorists from disparate disciplines assume that in tasks such as the IGT, where participants make repeated choices between two or more alternatives with differing outcomes, healthy individuals attempt to maximize net rewards over time (Samuelson, 1937; MacArthur and Pianka, 1966; Charnov, 1976; Rachlin et al., 1976; Damasio, 1994)^1^. When presented with the IGT—or any other novel choice task in which the contingencies of reward and punishment for each alternative are initially unknown—to maximize net rewards an individual must first *learn* the contingencies via trial and error. In a simple two-alternative choice task, learning is rapid and an exclusive preference may quickly develop, while in a more complex choice task, learning rate may be reduced and uncertainty increased. Preference at any given time depends on the individual's level of certainty of the relative contingencies. Behavioral economists traditionally distinguish three discrete categories of certainty—ambiguity, risk, and certainty (Knight, 1921; Ellsberg, 1961; Levy et al., 2010). However, in a choice task that requires learning, the boundaries between categories are not clear-cut; thus we argue that it is more helpful to conceptualize these classes as regions lying along a continuum of certainty (Figure 1). Initially an individual is completely uncertain, and frequently switches between alternatives to learn the contingencies—and preference can appear random or indifferent. But as the individual learns the approximate frequency and magnitude of rewards and punishers, preference will typically become biased toward alternatives with higher net rewards, and the individual may come exclusively to prefer the better alternative. Thus, an individual's location on the certainty continuum at the time preference is measured can critically impact the apparent “goodness” of their decision-making abilities.

Bechara et al. (1994) found that on the IGT, healthy participants appeared to exhibit this pattern of gradual learning over 100 trials and attained high net scores (an index of relative preference for good decks), while patients with VMPFC lesions generally failed to learn the contingencies, preferring alternatives with long-term net losses (Bechara et al., 1994; Damasio, 1994). This failure to maximize net rewards by VMPFC patients (and other clinical populations) was attributed by Bechara et al. (2000), Bechara and Damasio (2002) to atypical sensitivity to reward and/or punishment in these patients. More specifically, Bechara et al. (2000) hypothesized that poor IGT performance may result from three distinct types of decision-making deficit: hypersensitivity to reward; hyposensitivity to punishment; or myopia for the future—that is, insensitivity to delayed or infrequent events, whether they be rewards or punishers (Bechara et al., 2000, 2002; Bechara and Damasio, 2002).

Sensitivity to reward and sensitivity to punishment are terms used across multiple literatures but rarely formally defined. According to Davis and Fox (2008), individuals with high reward sensitivity “… are more prone to detect signals of reward in their environment, to approach with greater alacrity potentially rewarding stimuli, and to experience more positive affect (pleasure/reinforcement) when they are in situations with cues of reward.” (p. 43). Thus, sensitivity may be likened to an individual's subjective perception of, and “reactivity” to, a reward or punisher (e.g., a student may be more excited when given a $50 bill than a billionaire). This conception of sensitivity originates in reinforcement sensitivity theory (RST; Gray, 1970, 1991; Gray and McNaughton, 2000), in which reward sensitivity and punishment sensitivity are considered stable personality characteristics, associated with distinct neural substrates^2^.

In the IGT, sensitivity cannot be measured by analyzing behavioral metrics such as the number of responses to each deck, because each deck yields both rewards and punishers. For example, a high proportion of responses to Deck B (large, frequent rewards and large, infrequent punishers) may indicate either high sensitivity to reward or low sensitivity to punishment. Therefore, supplemental measures have been used to measure sensitivity. For instance, Bechara et al. (2000, 2002); Bechara and Damasio (2002) used a physiological measure (skin conductance response; SCR), inferring that, for example, a low-magnitude SCR (relative to control participants) in response to losing money during a trial was indicative of a low sensitivity to punishment. Other IGT studies (e.g., Suhr and Tsanadis, 2007; Buelow and Suhr, 2013) have utilized self-report measures of sensitivity (e.g., Carver and White, 1994; Torrubia et al., 2001).

A limitation of physiological and self-report approaches is that they don't measure the specific *dimensions* of sensitivity—such as sensitivity to the *frequency* and *magnitude* of rewards and punishers—that influence preferences in the IGT. To this end, it may be worthwhile adopting an alternative approach used in other literatures that also investigate decision making. In behavioral economics the perceived, or subjective value of a reward or punisher (referred to as its utility; Herrnstein, 1990; Glimcher and Rustichini, 2004) is assumed to differ from its physical, or objective value. This idea converges with the concept of sensitivity from RST: Individuals may differ in the extent to which they *scale* the physical properties of rewards and punishers to subjective perceptions. A variety of methods and models have been developed to quantify this scaling (e.g., Wearden, 1980; Herrnstein, 1990; Glimcher and Rustichini, 2004; Takahashi, 2005; McKerchar et al., 2010; Doyle, 2012). Some of these models were derived from the generalized matching law, an equation developed by operant psychologists (Baum, 1974; for an introduction see Poling et al., 2011). Advantages of the generalized matching law are that it mathematically formalizes sensitivity and offers a well-validated procedure to measure the individual dimensions of reward and punishment (e.g., frequency, magnitude, or delay). The generalized matching law has not previously been applied to investigating decision-making deficits in human participants; its use may potentially complement the physiological and self-report measures of sensitivity previously applied to IGT research by providing a more nuanced picture of decision-making processes.

In order to derive sensitivity estimates using the generalized matching law, operant psychologists utilize *concurrent-schedule* procedures (Bouton, 2007). In a concurrent-schedule task, participants choose between two or more responses (e.g., key presses), each of which yields a reward with a different probability. That is, two or more schedules of reward are presented to participants concurrently. The IGT can thus be considered a type of concurrent-schedule task. Nevertheless, despite the similarities, the decision-making literature has not yet drawn on the operant study of concurrent schedules to enhance understanding of how sensitivity contributes to performance on the IGT; the present study will be the first to do so.

In addition to lacking a direct measure of sensitivity, the IGT also experiences high inter-study variability. A recent review by Steingroever et al. (2013) revealed that the strength of the learning pattern in Bechara et al.'s (1994) healthy control group has rarely been matched by authors outside Bechara's laboratory. Rather, high inter-study variability was apparent, including a number of studies reporting very low net scores for healthy individuals. Such findings raise concerns about the interpretation of low scores as indicative of a decision-making deficit (Dunn et al., 2006). Further, deck-by-deck analyses of group data suggest that what was assumed to be a preference for the decks yielding higher long-term net gains may in fact reflect a preference for decks with a low frequency of losses (e.g., Wilder et al., 1998; Dunn et al., 2006; Lin et al., 2007, 2013; Steingroever et al., 2013). This tendency to avoid frequent losses (i.e., the *frequency-of-losses effect)* throws into question the assumption that healthy participants learn to maximize net rewards (Bechara et al., 1994).

An issue that may contribute to high inter-study variability, but which has rarely been discussed, is that the IGT procedure itself has been inconsistently implemented across studies (Areias et al., 2013). The majority of IGT researchers have devised proprietary implementations in which fundamental parameters—including task complexity, task instructions, and task length —often vary. Task complexity (equivalent to contingency discriminability in operant psychology; Davison and Jenkins, 1985) is determined by basic design parameters including the number of choice alternatives, the number of dimensions (e.g., valence, magnitude, frequency, or delay), the predictability of rewarding and/or punishing events, and the variability of reward/punisher magnitudes. Operant studies (e.g., Takahashi and Iwamoto, 1986; Hanna et al., 1992) suggest that participants' choice behavior may be affected even by subtle task variations (e.g., appearance, color, and spatial positioning of stimuli on the screen; labeling of decks; randomization of deck position and card order; changes in card appearance or color, printed feedback, and audio associated with wins or losses). Moreover, task instructions often include information about the reward/punishment contingencies, which can influence participants' initial level of certainty. Both operant (e.g., Horne and Lowe, 1993) and IGT (e.g., Balodis et al., 2006; Fernie and Tunney, 2006; Glicksohn and Zilberman, 2010) research has demonstrated that instructions can profoundly affect the ability of participants to learn the contingencies. In particular, a “hint” in Bechara's instructions (stating that some decks are better than others and that participants should avoid bad decks) has been shown to be critical to good IGT performance (Balodis et al., 2006; Fernie and Tunney, 2006; Glicksohn and Zilberman, 2010).

In addition to high inter-study variability in the IGT, Steingroever et al. (2013) found high inter-individual variability when net scores and deck preferences for individual participants (as opposed to group data) were examined in detail. Not only did individual net scores vary widely, but up to a third of healthy participants in some studies obtained scores low enough to be classified alongside VMPFC patients. Thus, it appears that the typical practice of aggregating individual participants, decks, and trials when analyzing IGT data may obscure important information, creating confusion in interpretation and perhaps leading one to believe in the fictitious average healthy participant.

Following Bechara et al.'s (2000) explanation of poor IGT performance in terms of atypical sensitivity to reward or punishment, and consistent with the view of sensitivity as a critical personality trait (Gray and McNaughton, 2000), it might be hypothesized that the high inter-individual variability in IGT performance is due to individual differences in reward sensitivity or punishment sensitivity in healthy participants. However, Steingroever et al. (2013) also found that individual learning trajectories did not typically resemble the gradual learning curve suggested by group data. While many participants established a stable preference for one or more decks during the allowed 100 trials, they did so at varying times, and final deck preferences often differed. Still other participants never established stable preferences, exhibiting high switching rates between decks and low net scores throughout the task.

This variability in learning rate may be an important contributor to high inter-individual variability: If some healthy individuals learn the task very slowly, then their net scores (based on all 100 trials) will be considerably lower than those who learn the task very quickly, resulting in a wide range of individual net scores. Thus, as some authors have noted (Dunn et al., 2006; Wetzels et al., 2010; Buelow et al., 2013; Ryterska et al., 2013), one cannot infer that a poor net score in the IGT is due to a decision-making deficit (i.e., atypical sensitivity), or a low rate of learning, or both.

In operant research, to control for individual differences in learning rate, a task will typically continue until participants have developed a stable preference according to some predefined stability criterion (see Sidman, 1960; Killeen, 1978; Baron and Perone, 1998). Data from earlier, learning trials are then discarded and analysis is only carried out on data from later, stable trials. In contrast, the IGT is typically fixed at 100 trials for all participants, and all data are analyzed—including data from early trials when participants were learning by trial and error (refer Figure 1). This is somewhat akin to evaluating the balance and coordination of a group of novice snowboarders by allowing them 10 attempts at negotiating an intermediate-level trail, and measuring the total number of times they fall over. This is effectively an assessment of learning rate—a better approach would be to first allow each participant to reach a predefined competency level (which will inevitably take a varying amount of time for each individual), before evaluating their performance on the intermediate trail. Indeed, several IGT studies have shown that when allowed more than 100 trials, many individuals who perform poorly during the first 100 trials are able to achieve good performance by Trial 200 (e.g., Fernie and Tunney, 2006; Buelow et al., 2013) or Trial 300 (Lin et al., 2013).

The present study comprised two experiments. In Experiment 1 we investigated factors other than sensitivity (i.e., poor task standardization and individual differences in learning rate) that may contribute to the high variability in IGT performance in healthy participants. In Experiment 2 we developed a novel card task based on human operant experimental procedures and applied the generalized matching law to derive behavioral estimates of sensitivity for the participants in Experiment 1 to investigate whether sensitivity differed between individuals categorized as good or poor decision makers on the IGT.

## Experiment 1

In Experiment 1 a 200-trial version of the IGT was administered to 50 participants, systematically replicating two recent studies that examined the effect of trial length on IGT performance (Buelow et al., 2013; Lin et al., 2013). In addition to increasing task length, we controlled task complexity and task instructions by implementing the IGT as closely as practicable to Bechara's original computer-based IGT (first described in Bechara et al., 1999)^3^. Operant procedures guided the analysis—we did not limit our analyses to group data, but also examined individual learning trajectories, allowing us to better capture individual differences in deck preferences. Moreover, similar to operant analyses, we established a stability criterion that allowed us to limit the analysis of net scores to stable data.

We hypothesized that group data in the first 100 trials would replicate Bechara et al.'s (1994, 1998, 1999) findings with healthy controls more closely than other studies that have introduced variations in instructions, procedure, and stimuli. Nevertheless, we predicted that individual net scores during the first 100 trials would be highly variable, and up to a third of healthy participants would perform as poorly as VMPFC patients. When given another 100 trials in which to learn the contingencies, however, we expected that mean net scores would improve and the majority of participants would develop stable preferences for one or both good decks. Thus, we hypothesized that individual differences in learning rate and deck preferences across 200 trials would contribute to high inter-individual variability and poor mean net scores.

### Method

#### Participants

In line with previous sample sizes reported by Bechara and colleagues (*N* ranging from 13 to 49; Bechara et al., 1994, 1998, 1999, 2000, 2002; Bechara and Damasio, 2002), we enrolled 50 young adults (20 males) aged from 17 to 32 years (*M* = 21.44; SD = 3.79) as participants in the current study. Participants were recruited in response to advertisements at the University of Auckland and were informed they would receive NZ$10 for completing the study, and could earn up to an additional NZ$20 depending on their performance in the “card games.” This was intended to encourage participant engagement (particularly in the more onerous tasks in Experiment 2); in actuality the design ensured all participants received the full NZ$30.

#### Experimental task

The IGT was based on the implementation in Version 0.12 of the Psychology Experiment Building Language test battery (PEBL; Mueller, 2009; Mueller and Piper, 2014). Mueller's version was modified to more closely replicate Bechara's computer-based IGT (Bechara et al., 1999)^4^ Instructions (see Supplemental Materials) were based on those provided by Bechara to Davison (2009) and similar to Bechara et al. (1999); notably, they included the following hint, previously shown to be critical to good IGT performance (Balodis et al., 2006; Fernie and Tunney, 2006; Glicksohn and Zilberman, 2010):

The only hint I can give you, and the most important thing to note is this: Out of these four decks of cards, there are some that are worse than others, and to win you should try to stay away from bad decks.

**Figure 2:**
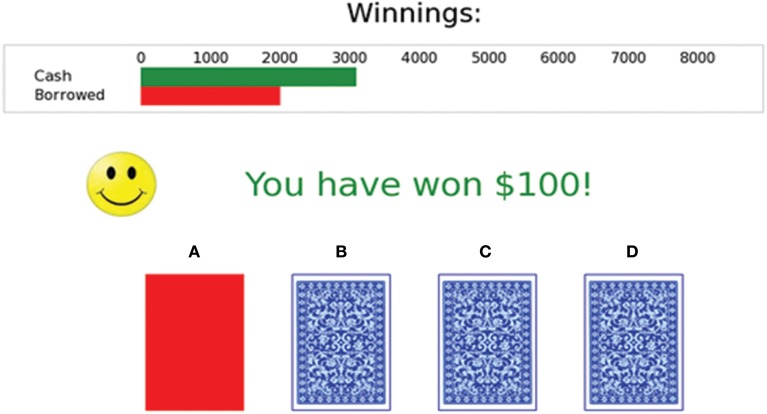
**Screen shot of the Iowa Gambling Task (modified PEBL implementation)**. The participant has just clicked on Deck A and received a $100 reward.

As argued in the Introduction, subtle variations in the experimental task may have an important influence on performance; therefore the IGT is described here in detail. A screen shot of the task is shown in Figure 2. Three differences distinguished this implementation from Bechara et al.'s (1999) version. First, the task was run for 200 trials instead of the standard 100 trials. Bechara's schedule only defined outcomes for 40 cards in each deck, so to ensure that a given deck in the current study would not run out of cards, the original schedule was repeated if a participant chose more than 40 times from a single deck. Second, instructions were presented on the computer screen rather than verbally to mitigate potential experimenter effects. Third, to promote task engagement, participants were told that their real-money winnings would be proportional to their play-money winnings in the IGT^5^.

Participants used the mouse to select a card from a deck. Following card selection, the card changed to either black or red while the outcome was displayed. Note that black and red cards did not necessarily correspond to wins or losses, but were arranged according to the schedule designed by Bechara et al. (1994, 2000). The message “You have won $” followed by the amount of the reward was then displayed alongside a smiley face, and a winning sound was played. If a penalty was also scheduled, it was displayed immediately after the winning sound; the phrase “But you also have lost $” was displayed alongside a sad face, and a losing sound was played. Sounds were identical to those in the implementation provided to our laboratory by Bechara (Davison, 2009). The inter-trial interval was approximately 2.5 s for reward-only trials, or 5 s when a reward was also followed by a penalty. Two bars were displayed at the top of the screen. The upper (green) bar displayed the amount of play money won during the game, and was updated appropriately after each card selection. The lower (red) bar displayed the amount of play money borrowed to play the game. If the participant's winnings fell below zero, a further loan of $2000 was automatically added to the red bar, and the green bar was reset appropriately. After participants had made 200 selections, they were informed of their net winnings (total winnings less the total amount borrowed) and the task ended.

#### Procedure

Informed consent was obtained in a manner approved by the University of Auckland Human Participants Ethics Committee. Participants performed all tasks alone in a quiet testing room. Experimental tasks were run on a Dell PC running Microsoft Windows XP. Stimuli were presented in full-screen mode on a Dell 22-inch LCD display at the native screen resolution of 1680×1050 pixels.

### Results

#### Group data

In line with standard IGT analytical approaches, we first analyzed data at the group level. In their seminal study, Bechara et al. (1994) introduced two summary statistics: mean number of selections from each deck over 100 trials, and mean net score (number of choices from good decks C and D minus number of choices from bad decks A and B) over 100 trials. Later analyses (beginning with Bechara et al., 2000) presented mean net score as a function of each 20-trial block (highlighting the learning curve in the IGT). To facilitate comparison with previous studies, we present similar analyses; however, as the present study used 200 trials, net scores are expressed as proportions (i.e., net score divided by the number of trials) rather than absolute numbers, and in some cases data are shown separately for Trials 1–100, Trials 101–200, and Trials 1–200. For inferential analyses we utilized ANOVAs as per the standard approach to IGT data analysis since Bechara et al. (1999).

Table 1 shows that mean net scores in the first 100 trials were similar to those found previously by Bechara and colleagues—particularly studies that utilized the computer-based IGT (Bechara et al., 1999) rather than the original IGT, which employed physical cards and facsimile money (Bechara et al., 1994). Mean net scores for Trials 1–100 in the present study were in the top 25% of the studies reviewed in Steingroever et al. (2013)^6^. Notably, the top-scoring study in Steingroever et al.'s review (North and O'Carroll, 2001) used the original computerized IGT supplied by Bechara. Thus, our results support the hypothesis that closer adherence to Bechara's experimental task and instructions would facilitate a closer replication of their results.

**Table 1:** **Mean net scores (expressed as proportions of number of trials) and aggregated deck preferences in Bechara et al.'s studies and in the present study**.

**Study**	***N***	**Trials**	**Mean Net score^c^**	**Mean proportion of choices**	
				**Good decks**	**Bad decks**
Bechara et al., 1994^a^	44	1–100	0.36	0.68	0.32
Bechara et al., 1998	21	1–100	0.24	0.62	0.38
Bechara et al., 1999^b^	13	1–100	0.28	0.64	0.36
Present study	50	1–100	0.24	0.62	0.38
		101–200	0.60	0.80	0.20
		1–200	0.42	0.71	0.29

a,b *Proportions were extrapolated from figures in the original papers; however, the estimated proportions for the 1994 and 1999 studies differ slightly from those reported by Steingroever et al. (2013)*.

c *Mean net score is mean proportion of choices from good decks (C and D) minus mean proportion of choices from bad decks (A and B). Proportions rather than whole numbers are used to allow comparison across different numbers of trials (multiply by 100 to compare with net scores from 100-trial IGT studies)*.

**Figure 3:**
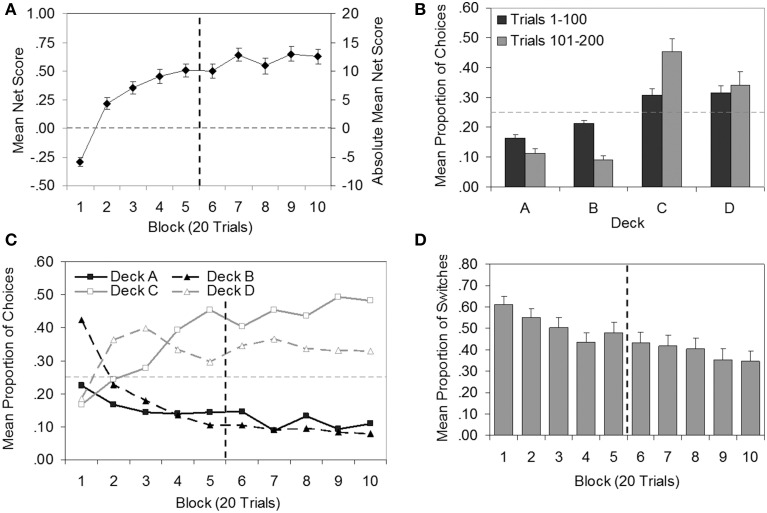
**Plots of group data (*N* = 50) for the Iowa Gambling Task. (A)** Mean net score by block (secondary axis displays absolute mean net score for comparison with previous literature). **(B)** Mean proportion of choices by deck for each 100-trial epoch. **(C)** Mean proportion of choices by block for each deck (error bars omitted for clarity). **(D)** Mean proportion of switches by block. Error bars show standard error of the mean. Vertical dashed line demarcates trials 1–100 and 101–200, where relevant. Horizontal dashed line indicates the level at which the number of choices from bad decks was equal to the number of choices from good decks **(A)**, or chance selection **(B,C)**.

In Figures 3A,B, standard graphical depictions of IGT data are presented. Figure 3A shows mean net score as a function of 20-trial blocks. To determine whether, on average, performance continued to improve after 100 trials, a repeated-measures ANOVA (Greenhouse-Geisser corrected) was carried out on net scores, which confirmed a significant effect of block [*F*_(5.86, 287.21)_ = 47.75, *p* < 0.001, η^2^_*p*_ = 0.49 ]. Significant linear [*F*_(1, 49)_ = 126.00, *p* < 0.001, η^2^_*p*_ = 0.72] and quadratic trends [*F*_(1, 49)_ = 69.64, *p* < 0.001, η^2^_*p*_ = 0.59 ] were found, supporting the visual impression from Figure 3A that performance improved over time and had a curvilinear shape, leveling out somewhat in later blocks in an asymptotic pattern. Planned comparisons indicated that net scores in Blocks 1–5 were significantly lower than net scores in Blocks 7, 9, and 10 (all *p* < 0.05), supporting the hypothesis that average IGT performance would improve if participants were given more trials in which to learn the task.

Figure 3B shows the total proportion of selections from each deck for all participants. Results are shown separately for Trials 1–100 and Trials 101–200 (referred to here as epochs). A repeated-measures ANOVA (epoch × deck; Greenhouse-Geisser adjusted) revealed a main effect of deck [*F*_(1.55, 76.15)_ = 19.20, *p* < 0.001, η^2^_*p*_ = 0.28] and an interaction between epoch and deck [*F*_(1.49, 72.81)_ = 20.55, *p* < 0.001, η^2^_*p*_ = 0.30]. *Post-hoc* pairwise comparisons showed that in each epoch, significantly more choices were made from the good decks C and D than the bad decks A and B (all *p* < 0.01). That is, on average, participants chose advantageously. The interaction between epoch and deck was reflected in different patterns of choice in the first and second epochs: Choice of Deck A (*p* < 0.001) and Deck B (*p* < 0.001) decreased significantly between the first and second epoch, while choice of Deck C increased significantly (*p* < 0.001). Preference for Deck D did not change across the two epochs (*p* = 0.39). Thus, the group data suggest that the improvement in performance in later trials was characterized by a shift in preference away from Decks A and B toward Deck C.

As several authors (e.g., Fernie, 2007; Buelow et al., 2013; Steingroever et al., 2013) have pointed out, the above analyses have limitations. The aggregation of data by block (Figure 3A) obscures preferences for individual decks, while aggregating by deck (Figure 3B) obscures the effects of time. Figure 3C is a more informative representation of the data, and is increasingly favored by IGT researchers. Figure 3C illustrates the trend toward good decks and away from bad decks over time (similar to Figure 3A), whilst also breaking down individual deck preferences (as in Figure 3B). Figure 3C indicates that, on average, participants learned to prefer the good decks (C and D) rather than the decks with lower frequencies of losses (B and D; cf. Steingroever et al., 2013). An examination of individual data further revealed that only 8% of the sample (Participants 9, 10, 45, and 46) exhibited a preference for Decks B and D over other decks in the first 100 trials, and only one (Participant 9) maintained this pattern of preference in the second 100 trials.

Steingroever et al. (2013) introduced a new descriptive analysis plotting the mean proportion of switches from one deck to another made during each block of trials. Figure 3D presents the corresponding data for the present study (though here each block is 20 trials in length whereas in Steingroever et al. each block was 10 trials). In contrast to most of the studies reviewed by Steingroever et al., mean switching appeared to decrease over blocks, suggesting that, on average, preferences stabilized as the task progressed.

#### Individual data

Group analyses may conceal important individual differences in behavior; therefore individual data were examined in more detail. Table 2 indicates that, as hypothesized, net scores showed high inter-individual variability, reflected in high standard deviations (relative to means) and ranges (when averaged across 100-trial epochs). The standard deviation was higher in Trials 101–200 than in Trials 1–100 because 44 participants improved their scores (with six obtaining perfect scores) but six participants actually obtained lower scores in the second epoch. In the first epoch, 30% of the sample scored as low as VMPFC patients according to Bechara et al.'s (2001) criterion, consistent with previous studies (Steingroever et al., 2013). However, in the second epoch, only 16% remained in this category. Thus, as predicted, running the IGT for 200 trials evidently provided participants with more opportunity to learn the contingencies and thus reduced the number of participants classified as poor decision makers. Increasing the number of trials to 200 did not require an unreasonable amount of time; participants took an average of 13.72 min (*SD* = 2.68) to complete the task.

**Table 2:** **Mean net scores (expressed as proportions of number of trials) in the present study, variability statistics, and proportions of participants satisfying criteria for impaired performance**.

**Trials**	**Net score**					**Proportion of participants impaired^a^**	
	***M***	***SD***	**Min**.	**Max**.	**Range**	**Net score <0.10**	**Net score <0.00**
1–100	0.24	0.24	−0.34	0.68	1.02	0.30	0.14
101–200	0.60	0.40	−0.48	1.00	1.48	0.16	0.08
1–200	0.42	0.30	−0.41	0.81	1.22	0.18	0.12

a *The criterion used to classify a participant as “impaired” was originally defined by Bechara et al. (2001), (p. 384) as an overall net score < 10 (i.e., net score < 0.10 in proportional terms), based on norms calculated from VMPFC patients. Most subsequent studies have also adopted this criterion; however Steingroever et al. (2013) used a stricter criterion of net score < 0.00 (in proportional terms), also shown here for comparison*.

We examined individual learning trajectories to establish whether individual differences in learning rate and deck preference contributed to the inter-individual variability evident when the data are aggregated into epochs of 100 trials (first two rows of Table 2). Initial visual inspection of individual participant profiles (see Supplemental Materials) suggested that, consistent with Steingroever et al.'s (2013) analysis, participants took varying amounts of time to develop strong preferences, with many failing to do so even by 200 trials. Moreover, different participants developed stable preferences for different decks, or pairs of decks.

To quantify these visual observations, a stability criterion (see Baron and Perone, 1998) was devised for the IGT. While no specific operant criterion was suitable for the IGT, an analogous approach was taken. Specifically, a participant was considered to show a strong preference for a single deck when the proportion of choices from that deck during a block was (a) at least 0.50 and (b) at least 0.25 greater than the proportion of choices from any other deck. For participants who did not prefer one particular deck, a strong preference for a pair of decks was assumed when (a) the sum of the proportion of choices from the two decks during a block was at least 0.75 and (b) the preference for each of the two decks differed by less than 0.25. Behavior was considered stable when the same preference was maintained for three consecutive blocks (i.e., 60 trials).

**Figure 4:**
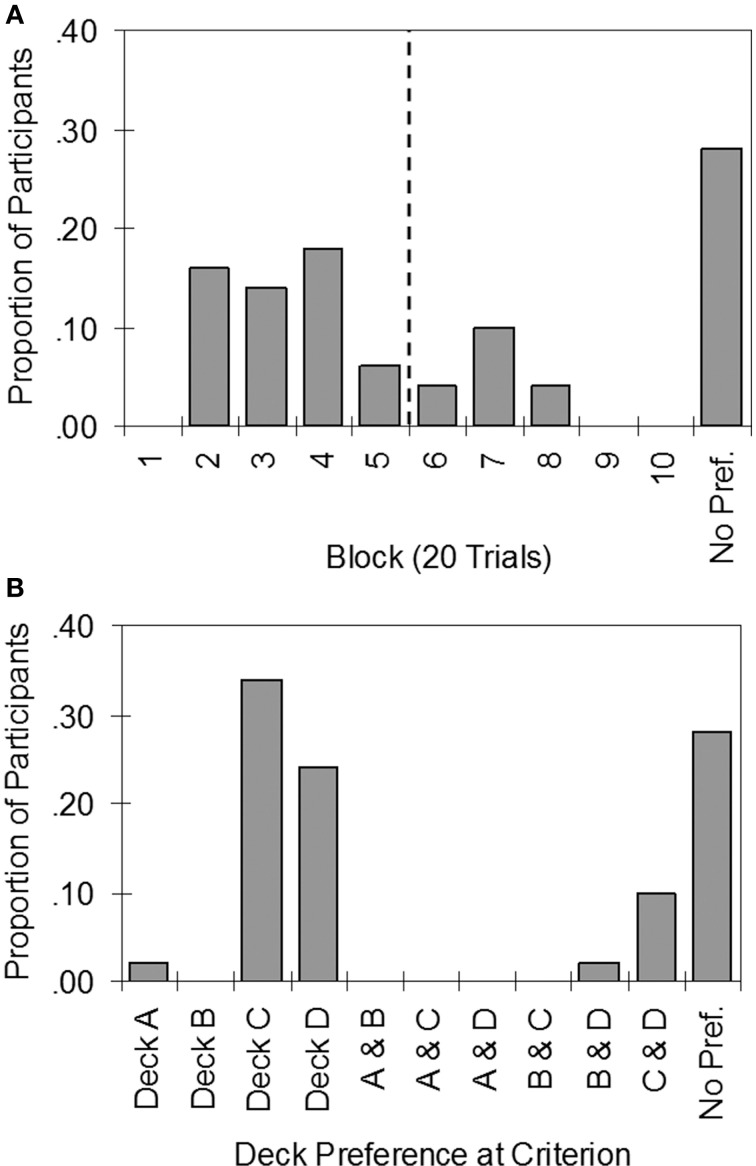
**Histograms summarizing the results of applying the stability criterion to individual data in the Iowa Gambling Task. (A)** Proportion of participants to reach the stability criterion by each block of trials (vertical dashed line demarcates trials 1–100 and 101–200). **(B)** Proportion of participants who preferred each deck or pair of decks. No Pref., No preference by the end of the task.

Figure 4A indicates that individual participants achieved stable preferences at varying times between the second and eighth block of trials. Only 54% of participants met the stability criterion by 100 trials, while 72% of participants had done so by 160 trials (however, note that 4% of those who met the stability criterion preferred bad decks). The results are consistent with the view that healthy individuals differ widely in learning rate, and that many require more than 100 trials to develop a strong preference. Indeed, 28% did not meet the stability criterion even after completing 200 trials. Note that the new stability criterion is stricter than Bechara et al.'s (2001) criterion (Table 2), which only classified 16% of participants as impaired.

Figure 4B summarizes preferences for each deck and each pair-wise combination of decks according to the stability criterion. Of those who developed strong preferences, almost all preferred the good decks: 34% of the sample preferred Deck C, 24% preferred Deck D, and 10% preferred both C and D approximately equally. There were two exceptions: One participant strongly preferred Deck A, whilst another preferred Decks B and D.

The individual differences in learning rate and deck preferences apparent in Figure 4 contributed to high inter-individual variability (Table 2) due to the influence of the low net scores achieved by many participants. For example, in Trials 1–100 half the sample: 46% (23 participants) did not learn to prefer the good decks, while 4% (2 participants) developed preferences for bad decks. To examine the effect on performance in Trials 1–100 of controlling these two factors, analysis was restricted to the 50% of participants who mastered the IGT within 100 trials (see first row of Table 3). Compared to the entire sample (first row of Table 2), the mean net score was considerably higher and variability lower, supporting the hypothesis that individual differences in learning rate and deck preference contribute to poor performance and high inter-individual variability in the standard 100-trial IGT.

**Table 3:** **Mean net scores (expressed as proportions of number of trials) and variability statistics in participants who developed stable preferences for the good decks (C and/or D) in the present study**.

**Trials**	**Net score**				
	***M***	***SD***	**Min**.	**Max**.	**Range**
1–100 (*n* = 25)	0.39	0.14	0.08	0.68	0.60
161–200 (*n* = 34)	0.87	0.17	0.35	1.00	0.65

In operant research, differences in learning rate are controlled by discarding early learning data from analysis and focusing on preferences after subjects have satisfied the stability criterion. Similarly, to obtain a more accurate impression of how well healthy participants perform on the IGT, early learning trials should be excluded. In the present study, 68% of participants had achieved stable preferences for good decks by Trial 160; therefore data for these participants in the final 40 trials of the 200-trial IGT were examined (second row of Table 3). The mean net score of participants at stability was 0.87, and 13 of the 34 participants achieved scores of 1.0, suggesting certainty. We conclude that, given sufficient time to learn the task, the majority of healthy participants are able to perform extremely well on the IGT. Nevertheless, 28% failed to develop a strong preference even after 200 trials—this was investigated further in Experiment 2.

### Discussion

Historically, IGT studies have not placed high importance on procedural details, evidenced by the wide variety of procedures used (Areias et al., 2013), and the tendency not to report details of the experimental task in method sections. In the present study we report evidence supporting the hypothesis that the variations in IGT task complexity and instructions found in the literature may contribute to inter-study variability. Specifically, our close replication of Bechara et al.'s (1999) computerized experimental task and instructions resulted in mean net scores in Trials 1–100 that were comparable to Bechara et al. (1998, 1999), in contrast to the relatively low scores reported in the majority of IGT studies reviewed by Steingroever et al. (2013).

Also like Bechara et al. (1994), but in contrast to many of the IGT studies reviewed by Steingroever et al. (2013), our group data for the first 100 trials showed no evidence of the tendency to avoid frequent punishers (i.e., the *frequency-of-losses* effect; Dunn et al., 2006; Lin et al., 2013). Rather, the descriptive data for our first 100 trials (see Figure 3B) resembled the pattern of strong preferences for Decks C and D in Bechara et al.'s (1994) control group, and only four individuals clearly preferred Decks B and D over other decks in the first 100 trials. It is unclear why the frequency-of-losses effect was not observed in the present study. We can only speculate that payment of participants (admittedly a departure from Bechara et al.'s original implementation) might have led them to be more averse to risky Deck B, although previous studies found that payment of real money in the IGT had little effect on performance (e.g., Bowman and Turnbull, 2003; Carter and Pasqualini, 2004; Fernie and Tunney, 2006). Thus, while it seems unlikely that in this case, payment affected performance, future work that systematically varies a variety of task factors is needed to determine precisely which aspects of the procedure are critical to achieving reliably high net scores^7^.

As hypothesized, mean net scores exhibited the high variability typically found in IGT studies, along with the common finding that many healthy participants (30% in the present study) perform as poorly as VMPFC patients in the standard 100-trial IGT (Steingroever et al., 2013). Also as predicted, increasing the number of trials greatly improved performance—in Trials 101–200 the number of participants who performed in the range of VMPFC patients was nearly halved to 16% of the sample. The improvements in performance after 100 trials replicated the findings of the few papers that have examined the effect of extending the number of trials (e.g., Fernie and Tunney, 2006; Buelow et al., 2013; Lin et al., 2013). Thus, our study adds to a growing chorus that the existing standard 100-trial IGT may be inappropriate for clinical assessment, as it classifies a disproportionate number of healthy participants as impaired.

Our novel analysis of individual data supported the hypothesis that the inter-individual variability in IGT net scores is likely attributable to individual differences in learning rate and, to some extent, to differing preferences for individual decks. When learning rate was controlled by adopting an operant approach—that is, restricting analysis to stable data from participants who met the stability criterion—inter-individual variability in net scores was reduced relative to the group as a whole. This observation suggests that the misclassification of many healthy individuals as impaired may reflect wide differences in individual learning rates. Indeed, while some participants required only 40 trials to develop stable preferences for the good decks, others required 160 trials. Moreover, contrary to Brand et al.'s (2007) assertion that most participants have a good idea of the contingencies by 40–50 trials, only 16% of our participants developed stable preferences by 40 trials. We suggest that preferences should be analyzed only when they have stabilized in the majority of participants, which appeared to take at least 160 trials in the present sample. Nevertheless, 160 trials was close to the limit of 200 trials, and it is possible that given more trials, a larger majority of participants would have mastered the task. Further work is required to clarify the appropriate absolute trial limit in the IGT that allows the majority of healthy individuals to develop stable preferences.

Note that due to the novelty of this analytic approach to the IGT, the basic stability criterion used herein was somewhat exploratory, and therefore our conclusions are tentative. For instance, the stability criterion employed here will not detect a late change in preference once an earlier preference has stabilized (examining the individual data, approximately seven participants showed late changes in preference after 60 stable trials). Given the wide range of learning rates across individuals, a higher absolute trial limit (e.g., 300 or 400 trials) would ideally be combined with a dynamic stability criterion—that is, where the task would halt once stability was reached in each participant. This would help prevent loss of engagement in the task, which we speculate may have led some of our participants to begin experimenting with other decks again after they had reached stability.

One might argue that applying a stability criterion and discarding learning trials from analysis defeats the purpose of the IGT—the designers of the IGT may have deliberately limited the task to only 100 trials because they intended the IGT to be an implicit measure of learning rate. The underlying assumption is that atypical sensitivity is likely to be associated with slower learning. However, slower learning is not necessarily due to atypical sensitivity—in the present study, 18% of healthy participants did not develop strong preferences for good decks until the second 100 trials. In Buelow et al.'s (2013) study, the equivalent figure was 26.5% (though a different calculation was used). Buelow et al. speculated that these slow learners may exhibit a different type of decision-making deficit, albeit less severe than those who never develop strong preferences. However, given that the slow learners represent approximately 20–25% of healthy participants, arguably the more parsimonious explanation is that this simply represents normal individual variability in learning rate (i.e., the upper tail of the distribution of learning rates). By disregarding learning data, we don't wish to imply that learning rate is not of interest in its own right. However, when examining learning rates, due to individual variability it is advisable to (1) analyze individual learning curves rather than the group learning curve, and (2) focus on behavior prior to attaining stable preference (given that stable preference is assumed to be a function of sensitivity, not learning *per se*).

The individual analyses further extend prior IGT work by classifying participants into sub-groups according to stable deck preference. Previous studies aggregated all participants and made conclusions based on group data. For example, both Buelow et al. (2013) and Lin et al. (2013) reported an overall preference for Deck D in Trials 101–200 (though by the third 100 trials in Lin et al.'s study, mean preference for C and D was almost equal), while our group data showed an overall preference for Deck C in the second 100 trials. However, focusing on group data can lead to an inaccurate perception of the preferences of healthy participants. Examination of our individual data revealed that participants fell clearly into three main sub-groups: About a third preferred Deck C; a quarter preferred Deck D, and 10% showed an approximately equal preference for Decks C and D. The small sample size in the present study restricts further statistical investigation of the characteristics and sensitivity profiles of these sub-groups. However, future work with larger samples might use these sub-groups to investigate several hypotheses. For example, high sensitivity to penalty magnitude may be associated with a preference for Deck C over Deck D (larger penalties); conversely, individuals with a high sensitivity to penalty frequency might prefer Deck D to Deck C (more frequent penalties).

The individual analyses raise a further question that cannot be addressed by the IGT data alone. Although in the second 100 trials only 16% of participants were classified as impaired by Bechara et al.'s (2001) criterion, by our stricter stability criterion 28% of participants failed to exhibit a stable preference for any particular deck or decks, even after completing 200 trials. These participants may have been particularly slow learners—perhaps they would have developed preferences for the good decks if allowed more than 200 trials (e.g., Lin et al., 2013). Alternatively, their weak preferences may be explained by atypical sensitivity to reward and/or punishment (Bechara et al., 2000). Lacking additional physiological (e.g., SCR) or self-report measures, the behavioral data in the IGT (i.e., preference for each deck) cannot be used to compute sensitivity measures. Therefore, Experiment 2 employed a novel operant card task to derive behavioral estimates of sensitivity in participants, facilitating a comparison of poor decision makers with good decision makers.

## Experiment 2

Operant researchers have traditionally investigated choice using concurrent-schedule procedures, in which subjects are presented with choices between two alternatives, one of which may provide rewards with a higher probability (responses are typically rewarded at variable intervals of time). Research has established that organisms ranging from fruit flies (e.g., Zars and Zars, 2009) to human beings (e.g., Takahashi and Shimakura, 1998) approximately *match* their preference for an alternative to the proportion of rewards received (once they have learned the contingencies, and behavior has stabilized). For example, if one alternative provides 75% of the rewards then approximately 70–75% of responses will be made to that alternative. This phenomenon, first quantified by Herrnstein (1961) and subsequently dubbed the matching law, allows researchers to derive an estimate of sensitivity to reward based on choice behavior.

To measure sensitivity, subjects typically complete a series of conditions, each with a different ratio of rewards arranged on the two alternatives. A linear function is then fitted to the log-transformed response ratios and reward ratios, with the slope of the line yielding a quantitative measure of sensitivity. Thus, sensitivity is defined as the degree to which the subject's relative choices change when the ratio of rewards changes. The supplemental materials contain an overview of this approach and provide the generalized matching law equation and its derivation (see Supplement C). For an introduction to the matching law, please see Poling et al. (2011). For more complete coverage, refer to Davison and McCarthy (1988).

A limitation of applying operant procedures to human participants is the large number of sessions normally required for each condition, which may lead to loss of engagement (e.g., Buskist et al., 1991). Davison and Baum (2000) introduced a new procedure in which a range of reward ratios is presented to participants as a series of components, or mini-conditions, within a single session. This approach was adopted by two recent human operant studies (Lie et al., 2009; Krägeloh et al., 2010) as an efficient way to measure sensitivity while keeping the task relatively short and thus maintaining participant engagement. While in Krägeloh et al. and Lie et al. only a single dimension was measured (sensitivity to reward frequency), the present study extends measurement to three additional dimensions: sensitivity to reward magnitude, sensitivity to punishment frequency, and sensitivity to punishment magnitude.

In Experiment 2, a sub-sample of participants completed our novel operant concurrent-schedule task—the Auckland Card Task (ACT). The generalized matching law (Baum, 1974; see Supplement C)^8^ was fitted to data, yielding behavioral estimates of sensitivity to reward magnitude, reward frequency, punishment magnitude, and punishment frequency. We hypothesized that sensitivity estimates would increase systematically as participants learned the contingencies; nevertheless, based on previous human operant research (see Kollins et al., 1997), we predicted that there would be considerable individual variability in sensitivity.

Utilizing participants' IGT performance from Experiment 1, we investigated whether individuals who persistently performed poorly in the IGT (i.e., those who did not prefer one or both good decks even after 200 trials) would exhibit measurable differences in sensitivity in the ACT (e.g., hypersensitivity to reward or hyposensitivity to punishment; Bechara et al., 2000) relative to those who performed well on the IGT.

### Method

#### Participants

Thirty of the 50 participants from Experiment 1 also completed the ACT (Participants 21–50; 15 males)^9^, a more-than-adequate sample size for the individual level analyses employed in human operant research (Lie et al., 2009; Krägeloh et al., 2010). The mean age of this sub-sample was 21.07 years (*SD* = 3.89).

#### Experimental task

**Figure 5:**
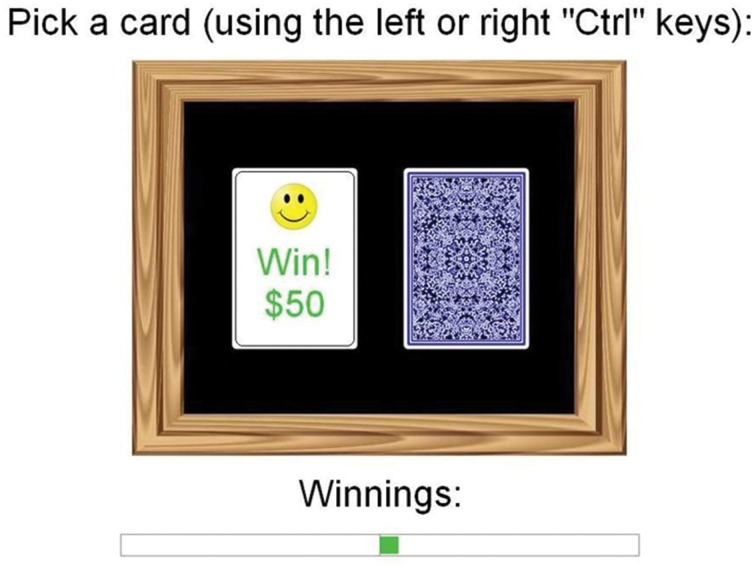
**Screen shot of the Auckland Card Task (Condition 1)**. The participant has just pressed the left control key and received a $50 reward.

The ACT was developed in Presentation (Version 16.1, Neurobehavioral Systems Inc., Albany, CA, USA). Like the IGT, the ACT required participants to choose between decks of cards containing both rewards and punishers. Aside from this superficial similarity, the ACT differed considerably from the IGT. Only two decks of cards were presented (Figure 5) and rewards/penalties were delivered probabilistically (see Table 4) rather than according to fixed schedules (e.g., the pre-ordered decks in the IGT). Participants were told that each deck contained hundreds of playing cards, and that some winning and losing cards had been shuffled into both decks. Like the IGT, screen instructions (see Supplemental Materials) included a hint as to strategy, which differed depending on the condition. For example, in Condition 1, the hint was:

Winning cards can be found in both decks, but one deck has MORE winning cards than the other. Both decks also contain an equal number of losing cards. In each round, to maximize your score in the time given, you'll first need to figure out which deck has more winning cards in it, then choose more often from that deck.

**Table 4:** **Summary of conditions in the Auckland Card Task**.

**Condition**	**Comp**.	**Rewards (Probability/Amount)**		**Penalties (Probability/Amount)**		**Net reward**	
		**Deck 1**	**Deck 2**	**Deck 1**	**Deck 2**	**Deck 1**	**Deck 2**
1. Reward frequency	1	0.25/$50	0.75/$50	0.50/$30	0.50/$30	$100	$600
	2	0.35/$50	0.65/$50	0.50/$30	0.50/$30	$200	$500
	3	0.65/$50	0.35/$50	0.50/$30	0.50/$30	$500	$200
	4	0.75/$50	0.25/$50	0.50/$30	0.50/$30	$600	$100
2. Reward magnitude	1	0.50/$25 v	0.50/$75 v	0.50/$30	0.50/$30	$100	$600
	2	0.50/$35 v	0.50/$65 v	0.50/$30	0.50/$30	$200	$500
	3	0.50/$65 v	0.50/$35 v	0.50/$30	0.50/$30	$500	$200
	4	0.50/$75 v	0.50/$25 v	0.50/$30	0.50/$30	$600	$100
3. Penalty frequency	1	0.50/$170	0.50/$170	0.75/$50	0.25/$50	$100	$600
	2	0.50/$170	0.50/$170	0.65/$50	0.35/$50	$200	$500
	3	0.50/$170	0.50/$170	0.35/$50	0.65/$50	$500	$200
	4	0.50/$170	0.50/$170	0.25/$50	0.75/$50	$600	$100
4. Penalty magnitude	1	0.50/$170	0.50/$170	0.50/$75 v	0.50/$25 v	$100	$600
	2	0.50/$170	0.50/$170	0.50/$65 v	0.50/$35 v	$200	$500
	3	0.50/$170	0.50/$170	0.50/$35 v	0.50/$65 v	$500	$200
	4	0.50/$170	0.50/$170	0.50/$25 v	0.50/$75 v	$600	$100

*Comp., Component (presented in random order). All conditions were dependent concurrent VI VI schedules with variable-interval timing and 2-s changeover delays. Conditions 1 and 2 were VI 8-s rewards; VI 20-s penalties. Conditions 3 and 4 were VI 20-s rewards; VI 8-s penalties. Reward and penalty schedules ran conjointly and independently of one another (see Critchfield et al., 2003). Conditions 2 and 4 imposed variable-magnitude rewards or penalties (indicated by “v”)*.

Participants made deck selections with their dominant hand by pressing the left or right control keys (the spacing of the control keys discouraged rapid alternation between decks). Most of the time, choosing a deck resulted in the brief display (200 ms) of a random playing card (from a standard 52-card deck) on top of the deck to simulate the top card being flipped over. However, occasionally (when determined by the dynamic scheduling algorithm; see Supplement E) a winning or losing card was displayed for 1000 ms and the bar graph was updated proportionately. If the score dropped below zero, the bar turned from green to red and extended to the left instead of to the right. A smiley face was displayed on winning cards above the amount won, and a “ding” sound was played. Losing cards featured a sad face and a “buzzer” sound. Both card decks were the same color; however, the deck color varied between conditions.

Although the instructions gave the impression that flipping cards faster would help participants win more money, in reality winning and losing cards were scheduled according to two separate concurrent variable-interval (VI) schedules. That is, a key press yielded a reward (or penalty) only when a variable interval of time had elapsed since the previous reward or penalty.

#### Experimental conditions

Each participant completed four different conditions (Table 4). Each condition allowed for behavioral estimates of sensitivity to one of four independent variables (reward frequency, reward magnitude, penalty frequency, and penalty magnitude) to be obtained. In each condition three dimensions were held constant and equal across the two decks, whilst the independent variable of interest was systematically varied across four components to provide data points for generalized matching law linear regressions. For example, in Condition 1 sensitivity to reward frequency was measured by varying the reward frequency ratio over four components, whilst reward magnitude remained constant and equal on both decks ($50), as did penalty frequency (0.5) and penalty magnitude ($30). In the first component of Condition 1, the reward frequency ratio was 1:3; that is, there were three times more rewards available on Deck 2 (15 rewards) than on Deck 1 (5 rewards). In the second component the ratio was approximately 1:2 (13 rewards on Deck 2 vs. 7 rewards on Deck 1). In the third and fourth components these ratios were reversed.

Within each condition, the four components were presented in random order, with a rest break between each component, during which the hint was repeated. Participants pressed the space bar when they wished to continue. Each component continued until the participant had received all scheduled rewards and penalties, or until 8 min had elapsed since the beginning of the component, whichever occurred first. The net reward for each component was identical in every condition; thus each participant was scheduled to win exactly the same amount in each condition (provided the component did not time out before they had received all the scheduled rewards and penalties).

In each condition, the reward and penalty schedules ran independently of one another (following Critchfield et al., 2003). To ensure that participants received the proportions of rewards and penalties that were arranged for each deck, dependent scheduling (Stubbs and Pliskoff, 1969) was used. Further details of the scheduling algorithms are provided in the supplemental materials (Supplement E). At the end of each condition participants were informed of their total play money winnings. A random amount was added to or subtracted from this total so participants wouldn't realize that, despite their efforts, they were winning exactly the same amount in each game (this was because identical net rewards were arranged for each condition).

#### Procedure

The ACT was presented after the IGT, and the four conditions (Table 4) were presented in counterbalanced order across participants^10^.

### Results

#### Group data

**Figure 6:**
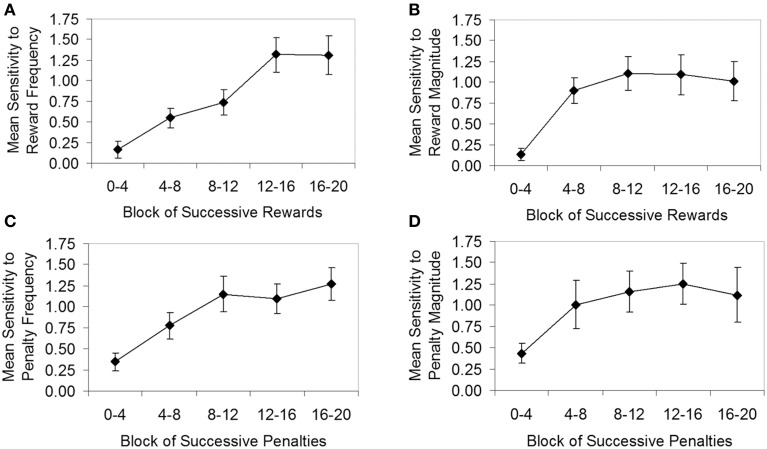
**Generalized matching law sensitivity (averaged across participants, *N* = 30) as a function of block for the four conditions of the Auckland Card Task. (A)** Condition 1, sensitivity to reward frequency; **(B)** Condition 2, sensitivity to reward magnitude; **(C)** Condition 3, sensitivity to penalty frequency; **(D)** Condition 4, sensitivity to penalty magnitude. Error bars show standard error of the mean.

Figure 6 follows the standard approach to analysing learning in Davison and Baum's (2000) experimental paradigm, showing mean sensitivity as a function of blocks of successive rewards or penalties. The graphs in Figure 6 can be considered analogous to Figure 3A for the IGT, but here the dependent variable is mean sensitivity rather than mean net score. The sensitivity estimates in Figure 6 are the averages of individual sensitivity estimates, derived by fitting the generalized matching law (see Supplement C) to the log response ratios and log reward/penalty ratios for each block across all four components. The approach taken follows that of Lie et al. (2009). Specifically, the log response ratio for each block was calculated based on all responses made during the block (e.g., from the start of the component to the fourth reward/penalty; from the fourth reward/penalty to the eighth reward/penalty, etc.). The log reward/penalty ratio for each block was based on all rewards/penalties received from the start of the component to the end of the block (e.g., from the start of the component to the fourth reward/penalty; from the start of the component to the eighth reward/penalty, etc.).

Unfortunately, participants did not always receive all the rewards and penalties scheduled by the task. At times, participants developed a strong or exclusive preference for one deck, which caused the dependent-scheduling algorithm to suspend further rewards or penalties on that deck, and eventually the component terminated after reaching its maximum time limit (8 min). Typically, it was not until the fourth or fifth block that participants began to exhibit a strong or exclusive preference. As a consequence, sensitivity estimates for some individuals in later blocks were very high and in some cases could not be calculated (i.e., where zero responses on one deck resulted in a zero denominator in the generalized matching law), resulting in some missing data (particularly in Condition 4). The missing data are evidenced in Figure 6 by larger standard errors in some later blocks.

Figure 6 shows that, as hypothesized, sensitivity estimates tended to be very low at the beginning of a component, but increased rapidly across blocks of rewards or penalties as participants learned the contingencies, approximately leveling out toward the last two blocks at the end of the component. To determine whether the increases in sensitivity apparent in Figure 6 were statistically significant, for each condition a repeated-measures ANOVA was carried out, with block as the within-subjects factor. In each condition, a significant main effect of block was found (Condition 1, *F* = 9.54, *p* < 0.001, η^2^_*p*_ = 0.26; Condition 2, *F* = 7.35, *p* < 0.001, η^2^_*p*_ = 0.25; Condition 3, *F* > 0.590, *p* < 0.001, η^2^_*p*_ > 0.25; Condition 4, *F* = 2.76, *p* < 0.05, η^2^_*p*_ = 0.15). In Condition 1, a significant linear trend [*F*_linear(1, 27)_ = 20.81, *p* < 0.001, η^2^_*p*_ = 0.44] was apparent, while Condition 2 showed significant linear [*F*_linear(1, 22)_ = 13.79, *p* < 0.01, η^2^_*p*_ = 0.39] and quadratic trends [*F*_quadratic(1, 22)_ = 13.04, *p* < 0.01, η^2^_*p*_ = 0.37], consistent with the concave-downward shape of Figure 6B. Conditions 3 and 4 were affected by missing data; nevertheless, significant linear trends were found for both Condition 3 [*F*_linear(1, 17)_ = 19.83, *p* < 0.001, η^2^_*p*_ = 0.54] and Condition 4 [*F*_linear(1, 16)_ = 5.48, *p* < 0.05, η^2^_*p*_ = 0.26].

#### Individual data

As expected, individual participants exhibited considerable variability in sensitivity, particularly in conditions in which reward or penalty magnitude was varied—in the final block of each condition, the lowest sensitivity estimate for an individual was -1.16 (obtained in Condition 2), whilst the highest sensitivity was 5.19 (in Condition 4)^11^. In order to provide reliable individual sensitivity values for the cross-task analysis (below), it was necessary to compute a measure of sensitivity for each individual that represented stable behavior in the ACT (i.e., deck preference after the contingencies have been learned). Piloting on 20 participants^9^ had already established that most participants developed a strong preference for the good deck after receiving around 8–12 rewards or penalties; therefore, no formal stability criterion was defined. Rather, Figure 6 was visually inspected to determine approximately when sensitivity reached stable levels in the group data (i.e., when minimal bounce and trend were apparent from block to block; see Baron and Perone, 1998). Based on these observations, we averaged the sensitivity estimates for each individual across the last two blocks in Condition 1, and across the last three blocks in Conditions 2–4.

#### Cross-task analysis

**Figure 7:**
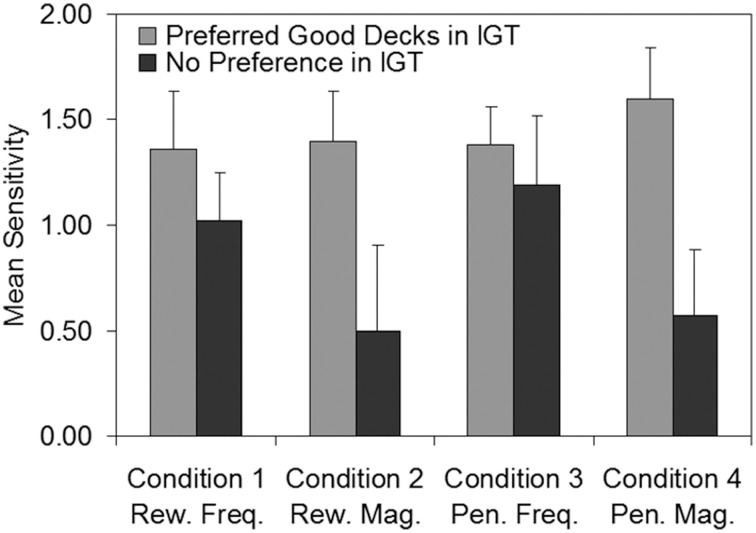
**Mean sensitivity estimates in the Auckland Card Task for participants who preferred the good decks (*n* = 19) in the Iowa Gambling Task vs. participants who developed no strong preference (*n* = 9)**. Error bars show standard error of the mean. Freq, Frequency; IGT, Iowa Gambling Task; Mag, Magnitude; Pen, Penalty; Rew, Reward.

To investigate whether non-learners in the IGT showed differences in sensitivity relative to those who mastered the IGT, we compared ACT sensitivities of persistent poor decision makers (participants who failed to meet the stability criterion in Experiment 1 after 200 trials; *n* = 9) with those of good decision makers (participants who learned to prefer Deck C and/or Deck D within 200 trials in the IGT; *n* = 19). Figure 7 shows mean ACT sensitivity for the two IGT sub-groups plotted as a function of each of the four ACT conditions. In consideration of the small and unequal sub-group sizes, two-tailed non-parametric Mann-Whitney *U*-tests were run. Good decision makers in the IGT exhibited significantly higher sensitivities to reward magnitude (*U* = 40.00, *p* = 0.035, *r* = −0.41) and penalty magnitude (*U* = 19.00, *p* = 0.025, *r* = −0.49) than poor decision makers. Thus, it appears that participants who developed no strong preferences on the IGT (and hence achieved low net scores) also exhibited lower sensitivity to the magnitude of both rewards and penalties in the ACT. There were no significant differences between the good and poor decision makers in their sensitivity to the frequency of rewards (*U* = 72.00, *p* = 0.643, *r* = −0.09) or penalties (*U* = 66.00, *p* = 0.595, *r* = −0.10).

### Discussion

Group data showed that, as expected, participants generally exhibited strong increases in preference (indexed by sensitivity) for the deck yielding higher net rewards as each component progressed, consistent with Krägeloh et al. (2010) and Lie et al. (2009). On average, preference stabilized after participants received approximately 8–12 rewards or penalties in most conditions.

The hypothesis that poor decision makers in the IGT would exhibit differences in sensitivity relative to good decision makers was also supported: Poor decision makers (i.e., those who failed to develop strong preferences for the good decks in the IGT after 200 trials) exhibited significantly lower sensitivity to the magnitudes (but not the frequencies) of rewards and penalties in the ACT. That is, while poor decision makers had little difficulty determining whether rewards or penalties occurred more often on one deck than the other in the ACT, they were poor at discriminating the average dollar amounts of rewards and penalties on each deck.

It is unlikely that low sensitivity to *reward* magnitude would have influenced deck choice in the IGT, as it was presumably trivial to discriminate which decks provided higher rewards (rewards in each deck were invariant in both frequency and magnitude). However, Dunn et al. (2006) noted that performance in the IGT depends primarily on participants' ability to avoid decks which impose higher penalties on average; therefore a low sensitivity to *punishment* magnitude may have influenced IGT performance. That is, participants who had difficulty determining which deck imposed larger penalties on average in the ACT would likely have had similar difficulties in the IGT, in which penalties also occurred relatively infrequently, irregularly, and (on two of the decks) with variable magnitudes.

Previous researchers offer four different hypotheses to explain poor performance on the IGT: Bechara et al. attribute poor IGT performance to hypersensitivity to reward, hyposensitivity to punishment, or myopia for the future (Bechara et al., 2000, 2002; Bechara and Damasio, 2002). Other authors have suggested poor performance is due to a preference for the decks with lower frequencies of losses (Decks B and D; Steingroever et al., 2013). Reconciling these explanations of poor IGT performance with the current findings presents a challenge—while IGT studies (e.g., Bechara et al., 2000, 2002) have sometimes used physiological instruments such as SCR to measure overall sensitivity to reward (and punishment), the operant procedure used here has allowed us to decompose sensitivity into the finer-grained levels of frequency and magnitude.

In the ACT, the frequency-of-losses effect would presumably be reflected in a higher sensitivity to the frequency of punishers in poor IGT performers. However, we found no such pattern in the ACT, and little evidence of a frequency-of-losses effect in the IGT. Hypersensitivity to reward would likely manifest in the ACT as higher sensitivity to reward frequency or magnitude in poor IGT performers, which was not observed. We found some evidence of hyposensitivity to punishment, as evidenced by lower sensitivity to punishment magnitude in poor IGT performers, but notably this did not extend to frequency. Our pattern of results is most compatible with Bechara et al.'s (1994) myopia for the future, which can arguably be formalized as a low sensitivity to the magnitudes of events (whether they are rewards or punishers) that occur infrequently. In the ACT, poor IGT performers exhibited lower sensitivity to the magnitudes of both rewards (Condition 2) and punishers (Condition 4). Both occurred infrequently due to the variable-interval scheduling.

This interpretation is tentative given the small and unequal sub-samples in the ACT-IGT cross-task analysis and the considerable individual differences in sensitivity. The high variability precludes employing the ACT in its current form as a clinical diagnostic tool to identify poor decision makers. To compete with the IGT, any new tool would have to be more effective than the IGT at dissociating impaired and non-impaired participants, and would require norming using large samples of healthy and clinical participants. Nevertheless, with further development the ACT may be useful in the experimental domain as it has enabled us to disambiguate sensitivity to magnitude and frequency, and demonstrate that lowered sensitivity to the magnitude of events, be they rewarding or punishing, is associated with poor IGT performance.

## General discussion

A coherent picture emerges from the two complementary experiments presented in this study. When a replica of Bechara et al.'s (1999) standard computer-based IGT (including the original instructions) was administered to healthy participants for an additional 100 trials, the majority (84%) achieved scores high enough to distinguish them from VMPFC patients, based on Bechara et al.'s (2001) criterion. When our stricter stability criterion was applied, it became apparent that most participants (68%) developed strong, stable preferences for good decks by Trial 160. Nevertheless, nearly a third (28%) failed to meet the stability criterion, and the choice behavior of these participants was characterized by frequent switching between decks throughout the task (rather than the strong preference for bad decks often exhibited by clinical participants; e.g., Bechara et al., 1994). Sensitivity measurements derived using the ACT suggested that the frequent switching shown by these participants may be related to a difficulty determining which decks impose penalties that are larger (on average), when those penalties vary in size and occur at unpredictable intervals.

While the atypical sensitivities found in these participants may represent a genuine decision-making deficit, it is also possible that the low sensitivity estimates might be a result either of very slow learning or some other confound; for example, loss of engagement (i.e., poor attention and motivation; Dunn et al., 2006) or inappropriate conscious strategy (e.g., risk appetite/aversion or superstitious behaviors; Skinner, 1948; Dunn et al., 2006). Without normed data on how many trials are required for IGT mastery (see Experiment 1 Discussion), we cannot rule out the possibility that some or all of these participants simply required more trials to reach stability.

Alternatively, the low sensitivity may have been due to loss of engagement in the task. Poor attention to the contingencies of reward and punishment is a documented problem in human operant research (Kollins et al., 1997), perhaps because operant tasks typically require large numbers of responses in order to derive reliable estimates of sensitivity. In the present study, participants may have found the ACT particularly tedious in comparison to the IGT, which provided rewards for every response. Thus, poor attention may have led to the near-zero (reflecting equal preference for both alternatives) sensitivity estimates in some individuals in the ACT. Similarly in the IGT, poor decision makers may not have attended properly to the reward and punishment contingencies. Nevertheless, from anecdotal observations, poor decision makers expressed apparently genuine disappointment and frustration at their low (or negative) winnings in the IGT, suggesting they were not inattentive. Additionally, in the ACT the mean sensitivity to reward and punishment *frequencies* in poor decision makers was not significantly different from that of good decision makers, suggesting attention to task at least in Conditions 1 and 3.

A second potential confound in concurrent-choice tasks such as the ACT and IGT is the development of inappropriate strategies or superstitious behavior. In the ACT this may be reflected in negative sensitivity (reflecting a preference for the poorer alternative), while in the IGT it may manifest in a preference for “risky” decks (Dunn et al., 2006). This confound may be mitigated in human participants with careful use of instructions; however, it can be difficult to strike the appropriate balance between instructions that give away too much information about the contingencies (leading to rapid learning and exclusive preference for the better alternative); or too little information (leading to no strong preference). The powerful influence of instructions on performance highlights the importance of standardizing instructions in the IGT, in which even healthy participants perform poorly unless the instructions specifically urge them to “… stay away from bad decks” (e.g., Balodis et al., 2006).

Notwithstanding the potential impact of confounding factors, the present study narrows the focus for future investigation of poor IGT performance in healthy participants: What type of deficit could give rise to difficulties in tracking the average size of punishers on each deck? Brand et al. (2007) found that performance in later trials in the standard 100-trial IGT was correlated with measures of executive performance; could low sensitivity to reward and punisher magnitudes reflect an executive deficit, rather than an affective decision-making deficit? Future researchers may wish to consider screening participants for issues such as dyscalculia (see Butterworth et al., 2011), and carrying out on poor decision makers additional post-study measures that probe numeracy abilities.

In conclusion, while the IGT has firmly established itself as the standard for studying decision making, and is widely used in both experimental and clinical settings, we offer three recommendations for its future application. First, to mitigate inter-study variability it is important that the task is properly standardized; that is, researchers should only use Bechara et al.'s (1999) original experimental task and instructions, or a close replica thereof. Second, to control for individual differences in learning rate that contribute to inter-individual variability, the task should be continued for a minimum of 200 trials (though more work is needed to determine the optimal limit), and only stable data should be analyzed (early trials reflecting trial-and-error learning are unreliable). Third, IGT analyses should not be limited to aggregated data (i.e., participants, decks, and trials)—important insights may potentially be gained from analysis at more detailed levels. Finally, while the ACT task used here has limitations, a cross-disciplinary approach, in which methods and models from behavioral economics and operant psychology are leveraged, may have potential in advancing the study of human decision-making deficits—particularly in its ability to quantify sensitivity and break it down into dimensions such as frequency and magnitude.

### Conflict of interest statement

The authors declare that the research was conducted in the absence of any commercial or financial relationships that could be construed as a potential conflict of interest.

## Footnotes

^1^There is evidence that organisms do not invariably maximize (reviewed in Herrnstein, 1997); however, it is outside the scope of this paper to examine the validity of the maximization assumption.

^2^Personality has been likened to “behavioral state,” defined as a distillation of an individual's previous history of reinforcement and punishment (Davison, 1998).

^3^We consider only the widely-used ABCD version of the Iowa Gambling Task and do not address the *A*′*B*′*C*′*D* and *E*′*F*′*G*′*H* variants introduced by Bechara et al.'s (2000).

^4^The modified IGT used in this study has been adopted as the default IGT implementation in PEBL Version 0.14 and can be downloaded from http://pebl.sourceforge.net/

^5^The weight of evidence suggests that healthy participants who receive real money in the IGT do not differ significantly in performance from those who do not (Bowman and Turnbull, 2003; Carter and Pasqualini, 2004; Fernie and Tunney, 2006; but see Vadhan et al., 2009).

^6^Note that one of the studies in Steingroever et al.'s review (Overman et al., 2004) administered 200 trials instead of the standard 100, so it is questionable whether it should have been included. Excluding Overman et al., the mean net scores in Trials 1–100 of the present study are the seventh highest (alongside Bechara et al., 1998) of the 31 studies reviewed.

^7^Unfortunately few clues can be found in previous published IGT studies, which generally provide little to no detail about the experimental task. However, we note that in some studies (e.g., Chiu and Lin, 2007; Fernie, 2007) the spatial positions of the card decks were randomized to control location bias, so an interesting initial avenue of research may be to investigate the effects of spatial position.

^8^The generalized matching law is an empirical model of behavior which, in contrast to cognitive models such as the expectancy-valence model (Busemeyer and Stout, 2002), makes no assumptions regarding underlying cognitive processes.

^9^ Participants 1–20 completed a pilot version of the ACT, during which the contingencies and instructions were adjusted to optimize reliability and validity. Because of the variation in task factors during piloting, data from Participants 1–20 were excluded from ACT analyses.

^10^Following Conditions 1–4 all participants also completed two additional conditions (Conditions 5 and 6) in which reward and penalty magnitudes were further manipulated; however, these conditions were irrelevant to the hypotheses examined in the current study and were therefore excluded from analysis.

^11^In comparison, Lie et al. (2009) obtained individual sensitivity values ranging from -0.02 to 2.17. Traditional animal operant studies of choice generally yield sensitivity estimates between about 0.80 and 1.00 (Baum, 1979; Taylor and Davison, 1983).
