# Supplementary material for: Decision making in healthy participants on the Iowa Gambling Task: new insights from an operant approach
Source: Front Psychol. 2015 Apr 7;6:391. doi: 10.3389/fpsyg.2015.00391 (PMC4387474; doi:10.3389/fpsyg.2015.00391)
Supplement: Supplementary file 1 [file DataSheet1.DOC]

**Supplemental Materials**

**Supplement A: Screen Instructions for Iowa Gambling Task (Experiment 1)**

In front of you on the screen, there will be 4 decks of cards: A, B, C, and D. When we begin the game, I want you to select one card at a time by clicking on a card from any deck you choose. Each time you select a card, the computer will tell you that you won some money. I don't know how much money you will win. You will find out as we go along. Every time you win, the green bar gets bigger. Every so often, when you click on a card, the computer will tell you that you won some money as usual, but then it will say that you lost some money as well. I don't know when you will lose or how much. You will find out as we go along. Every time you lose, the green bar gets smaller. Click the mouse to continue.

You are absolutely free to switch from one deck to the other at any time, and as often as you wish. The goal of the game is to win as much money as possible and avoid losing as much money as possible. You won't know when the game will end. Simply keep on playing until the computer stops. I am going to give you $2000 of credit, the green bar, to start the game. The red bar is a reminder of how much money you borrowed to play the game and how much money you have to pay back before we see whether you won or lost. Click the mouse to continue.

The only hint I can give you, and the most important thing to note is this: Out of these four decks of cards, there are some that are worse than others, and to win you should try to stay away from bad decks. No matter how much you find yourself losing, you can still win the game if you avoid the worst decks. Also note that the computer does not change the order of the cards once the game begins. It does not make you lose at random, or make you lose money based on the last card you picked. If you have any questions, please ask the researcher now. Otherwise, click the mouse to begin.

**Supplement B: Individual Deck Preferences for Iowa Gambling Task (Experiment 1)**

|  |  |
| --- | --- |
|  |  |
|  |  |
|  |  |
|  |  |
|  |  |
|  |  |
|  |  |
|  |  |
|  |  |
|  |  |
|  |  |
|  |  |
|  |  |
|  |  |
|  |  |
|  |  |
|  |  |
|  |  |
|  |  |
|  |  |
|  |  |
|  |  |
|  |  |
|  |  |

**Supplement C: Generalized Matching Law Background & Equations (Experiment 2)**

In the 1960s, operant researchers studying two-alternative concurrent schedules of reinforcement discovered that, on average, animals tended to *match* their responding on each alternative to the proportion of reinforcers they received on each alternative (Herrnstein, 1961). For instance, if the left key provides reinforcement 25% of the time and the right key 75% (a left:right ratio of 1:3), then over the course of an entire session, approximately 25-30% of a pigeon’s responses will be emitted to the left key and 70-75% to the right. This seminal finding is known as the matching law*.* The relation is termed a law because it has subsequently been found to be both consistent and general, having been demonstrated in a diverse range of animals (Davison & McCarthy, 1988). The matching law can be expressed mathematically as:

| $\frac{B_{1}}{B_{1}+B_{2}}=\frac{R_{1}}{R_{1}+R_{2}},$ | (1) |
| --- | --- |

where *B_x_* = the number (or rate) of responses on Alternative *x* and *R_x_* = the number (or rate) of reinforcers on Alternative *x*. The equation is more commonly expressed in its ratio form, obtained after algebraic rearrangement:

| $\frac{B_{1}}{B_{2}}=\frac{R_{1}}{R_{2}}.$ | (2) |
| --- | --- |

However, organisms do not match response rate precisely to physical reinforcer rate, but rather to subjective reinforcer rate. If it is assumed that physical reinforcer rate is scaled to subjective reinforcer rate according to a power law, then it is a simple matter to incorporate a scaling parameter *s* into the matching law:

| $\frac{B_{1}}{B_{2}}=\frac{R_{1}^{s}}{R_{2}^{s}}=\left(\frac{R_{1}}{R_{2}}\right)^{s}.$ | (3) |
| --- | --- |

Equation 3 is inadequate in itself to describe real-world choice, however, as it lacks an error term to account for any constant proportional preference for one alternative over the other that is not explained by differences in reinforcer rate. Such a bias toward one alternative is due to uncontrolled independent variables (IVs), and may come about in an operant experiment, for example, if one key is marginally easier to peck than the other key, or if the pigeon prefers the brightness or colour of one key over the other. To account for bias, the rightmost term of Equation 3 can be multiplied by a constant *b* to yield:

| $\frac{B_{1}}{B_{2}}=b\left(\frac{R_{1}}{R_{2}}\right)^{s}.$ | (4) |
| --- | --- |

Equation 4 is known in the operant literature as the generalized matching law (GML). The GML was initially proposed by Baum (1974; but foreshadowed by Lander & Irwin, 1968; Staddon, 1968) as a purely empirical description to account for experimental data that deviated from strict matching (Equation 1). In the years following the formulation of the matching law, it became apparent that strict matching was the exception rather than the rule, and Equation 4 was devised to account for three common deviations from strict matching. The most consistently found deviation is undermatching, which occurs when preference is less extreme than predicted by strict matching (i.e., *s* < 1). The other deviations documented by Baum were overmatching (*s* > 1) and bias (described above).

The GML was subsequently extended to accommodate other IVs (Davison & McCarthy, 1988). Thus in the most general form of the GML, the reinforcer-rate variable *R* is replaced by *X*:

| $\frac{B_{1}}{B_{2}}=b\left(\frac{X_{1}}{X_{2}}\right)^{s},$ | (5) |
| --- | --- |

where *X* may be reinforcer rate (*R*), magnitude (*M*), immediacy (*I*), quality (*Q*), and so forth.

To obtain an estimate of sensitivity using the GML, the IV of interest is varied systematically over several conditions to yield a range of IV ratios (e.g., reinforcer rate ratios) and corresponding dependent variable (DV) ratios (response rate ratios). In animal studies, each condition will typically continue for many daily sessions until the subject has learned the task; that is, until they have developed a stable preference for one alternative or the other according to some stability criterion (Baron & Perone, 1998).

The IV and DV ratios from the last few sessions following stability are then log transformed, and the line of best fit is calculated using linear regression. A log transformation of Equation 5 results in the following equation:

| $\log\left(\frac{B_{1}}{B_{2}}\right)=s\log\left(\frac{X_{1}}{X_{2}}\right)+\log b.$ | (6) |
| --- | --- |

The slope of the linear regression (*s*) provides a quantitative estimate of sensitivity, and the intercept (log *b*) provides an estimate of bias. Thus Equation 6 yields an objective measure of sensitivity to the IV; that is, the degree to which preference changes when there is a change in reinforcer or punisher rate, magnitude, delay, and so on. See Poling, Edwards, Weeden, and Foster (2011) for an introduction to the matching law.

**Supplement D: Screen Instructions for Auckland Card Task (Experiment 2)**

**Introduction.** You will play a series of six different card games (three now, and three in the second session). Each game takes about 15-25 minutes, and the computer will tell you when the game is finished. Please notify the researcher when you complete each game so that the next game can be set up. Please press the space bar for instructions...

You will be presented with two virtual decks of playing cards. Each deck contains hundreds of cards. Shuffled into each deck are some WINNING cards, which will add money to your overall score, and some LOSING cards, which will subtract money from your score. One deck will always be better than the other, and your goal is to maximize your winnings by learning which deck is better.

Each game has four rounds, and you'll be given a chance to rest between each round. The good deck may change from round to round, so always attend closely to the winning and losing cards you receive. These cards will vary a lot, so at the start of each round you'll need to sample the cards from each deck until you can figure out which deck is better.

In each game you will be given a hint (which is repeated during rest breaks). It's important that you read and understand the hint, as it will provide you with a strategy to maximize your winnings. Please press the space bar to continue...

This first game is a quick practice game to get you familiar with the task. There are only two rounds, the decks contain only winning cards, and your score will not count toward your total winnings.

During the game, press the left "Ctrl" key on the keyboard to choose a card from the left deck, or the right "Ctrl" key to choose from the right deck. You are free to switch from one deck to the other at any time. Please use only your dominant hand to respond - do NOT use both hands.

If you have any questions, feel free to ask the researcher now or at any time during the practice game. Please press the space bar to continue...

**Hint for Practice Game.** In this practice game, winning cards can be found in both decks, but one deck has HIGHER winning dollar amounts (on average) than the other. In each round, to maximize your score in the time given, you'll first need to figure out which deck has the higher winning cards (but note the amounts vary), then choose more often from that deck.

**Hint for Condition 1.** Winning cards can be found in both decks, but one deck has MORE winning cards than the other. Both decks also contain an equal number of losing cards. In each round, to maximize your score in the time given, you'll first need to figure out which deck has more winning cards in it, then choose more often from that deck.

**Hint for Condition 2.** Winning cards can be found in both decks, but one deck has HIGHER winning dollar amounts (on average) than the other. Both decks also contain an equal number of losing cards. In each round, to maximize your score in the time given, you'll first need to figure out which deck has the higher winning cards (but note the amounts vary a lot), then choose more often from that deck.

**Hint for Condition 3.** High winning cards can be found in both decks, but there are also many losing cards. One deck has MORE losing cards than the other. In each round, to maximize your score in the time given, you'll first need to figure out which deck has more losing cards in it, then choose less often from that deck, only occasionally checking it for winning cards.

**Hint for Condition 4.** High winning cards can be found in both decks, but there are also many losing cards. One deck has HIGHER losing dollar amounts (on average) than the other. In each round, to maximize your score in the time given, you'll first need to figure out which deck has the higher losing cards (but note the amounts vary a lot), then choose less often from that deck, only occasionally checking it for winning cards.

**Supplement E: Scheduling of Contingencies in Auckland Card Task (Experiment 2)**

In each condition of Experiment 2, the reward and penalty schedules ran independently of one another (a procedure adopted from Critchfield, Paletz, MacAleese, & Newland, 2003). If a reward and a penalty were scheduled on the same deck at the same time, the order in which they occurred was determined randomly. Further, in each condition one concurrent schedule was rich (high overall frequency of outcomes) and the other was lean (low overall frequency of outcomes). The independent variable (IV) of interest was arranged according to the rich schedule. For example, in Conditions 1 and 2, reward frequency and magnitude, respectively, were the IVs of interest, and rewards were scheduled according to a concurrent variable-interval (VI) 8-s VI 8-s schedule (rich), while penalties were delivered on a concurrent VI 20-s VI 20-s schedule (lean). However, in Conditions 3 and 4 (in which penalty frequency and magnitude, respectively, were the IVs of interest) penalties were presented on a concurrent VI 8-s VI 8-s schedule, while rewards were presented on a concurrent VI 20-s VI 20-s schedule. In all conditions, twice as many outcomes were available on the rich schedule than on the lean schedule (e.g., in Condition 1, there were 20 rewards and 10 penalties in each component). The order in which Conditions 1-4 were presented was counter-balanced across participants.

In all conditions, to derive the lengths of the variable intervals between scheduled outcomes (rewards or penalties), the scheduling algorithm calculated a random number between 1 and 2*x* – 1, where *x* = mean interval (e.g., if the mean interval was four seconds, a random interval between 1 and 7 seconds would elapse before the next reward or penalty became available). Where variable magnitudes were used (Conditions 2 & 4), the magnitudes of rewards and penalties were derived from an arithmetic progression of the form: *x*, 3*x*, 5*x*, 7*x*, and so forth, where *x* = mean magnitude of outcome / total number of outcomes (see Elliffe & Alsop, 1996). For example, in Component 1 of Condition 2, the mean magnitude of rewards on Deck 1 was $25 and ten rewards were scheduled; therefore the ten rewards (which occurred in random order) had the magnitudes: $3, $8, $13, $18, $23, $28, $33, $38, $43, and $48 (note that magnitudes were rounded to whole dollar amounts). In contrast, the mean reward magnitude on Deck 2 was $75 and thus the ten rewards on Deck 2 had the magnitudes $8, $23, $38, $53, $68, $83, $98, $113, $128, and $143.

To try and ensure that participants received the proportions of rewards and penalties that were arranged for each deck, dependent scheduling (Stubbs & Pliskoff, 1969) was used. To implement dependent scheduling, rewards (or penalties) were scheduled according to a single base VI schedule, and then allocated either to the left or the right deck probabilistically according to the arranged contingencies. For example, in the first component of Condition 1, rewards were scheduled according to a base VI 4-s schedule, and then allocated to the left deck with a probability of .25, or to the right deck with a probability of .75. In addition, a two-second changeover delay (COD) was employed (Herrnstein, 1961). The COD ensured that whenever a participant switched from one deck to the other, a reward (or penalty) would not occur until at least two seconds had passed since changing over. CODs are common practice in concurrent-schedule procedures, and prevent changeover behavior from being adventitiously reinforced or punished, which might contribute either to weak preferences or to extreme preferences, respectively.

**Supplement F: References for Supplemental Materials**

Baron, A., & Perone, M. (1998). Experimental design and analysis in the laboratory study of human operant behavior. In K. A. Lattal & M. Perone (Eds.), *Handbook of research methods in human operant behavior* (pp. 45-91). New York, NY: Plenum.

Critchfield, T. S., Paletz, E. M., MacAleese, K. R., & Newland, M. C. (2003). Punishment in human choice: Direct or competitive suppression? *Journal of the Experimental analysis of Behavior*, *80*(1), 1-27.

Davison, M., & McCarthy, D. (1988). *The matching law: A research review*. Hillsdale, NJ: Erlbaum.

Elliffe, D., & Alsop, B. (1996). Concurrent choice: Effects of overall reinforcer rate and the temporal distribution of reinforcers. *Journal of the Experimental Analysis of Behavior*, *65*(2), 445-463.

Herrnstein, R. J. (1961). Relative and absolute strength of response as function of frequency of reinforcement. *Journal of the Experimental Analysis of Behavior, 4*, 267–272.

Lander, D. G., & Irwin, R. J. (1968). Multiple schedules: Effects of the distribution of reinforcements between components on the distribution of responses between components. *Journal of the Experimental Analysis of Behavior*, *11*(5), 517-524.

Poling, A., Edwards, T. L., Weeden, M., & Foster, T. M. (2011). The matching law. *The Psychological Record, 61*, 313–322.

Staddon, J. E. R. (1968). Spaced responding and choice: A preliminary analysis. *Journal of the Experimental Analysis of Behavior*, *11*(6), 669-682.

Stubbs, D. A., & Pliskoff, S. S. (1969). Concurrent responding with ﬁxed relative rate of reinforcement. *Journal of the Experimental Analysis of Behavior 12*(6), 887–895.
